# Evaluation of trypanocidal drugs used for human African trypanosomosis against *Trypanosoma lewisi*

**DOI:** 10.1051/parasite/2013038

**Published:** 2013-10-22

**Authors:** Mariette Dethoua, Romaric Nzoumbou-Boko, Philippe Truc, Sylvie Daulouède, Pierrette Courtois, Bruno Bucheton, Gérard Cuny, Silla Semballa, Philippe Vincendeau

**Affiliations:** 1 Laboratoire de Parasitologie, BP 43, UMR 177 IRD CIRAD Université de Bordeaux 33076 Bordeaux Cedex France; 2 Institut de Recherche pour le Développement, Unité Mixte de Recherche 177 IRD-CIRAD, Campus International de Baillarguet, TA A17/G 34398 Montpellier Cedex 5 France

**Keywords:** Trypanosome, *Trypanosoma lewisi*, Trypanocidal drugs, Fexinidazole, Atypical trypanosomiasis

## Abstract

Trypanosomes from animals are potential pathogens for humans. Several human cases infected by *Trypanosoma lewisi*, a parasite of rats, have been reported. The number of these infections is possibly underestimated. Some infections were self-cured, others required treatment with drugs used in human African trypanosomosis. An *in vitro* evaluation of these drugs and fexinidazole, a new oral drug candidate, has been performed against *T. lewisi* in comparison with *T. brucei gambiense*. All have comparable activities against the two parasites. Suramin was not effective. *In vivo*, drugs were tested in rats immunosuppressed by cyclophosphamide. The best efficacy was obtained for fexinidazole, and pentamidine (15 mg/kg): rats were cured in 7 and 10 days respectively. Rats receiving nifurtimox-eflornithine combination therapy (NECT) or pentamidine (4 mg/kg) were cured after 28 days, while melarsoprol was weakly active. The identification of efficient drugs with reduced toxicity will help in the management of new cases of atypical trypanosomosis.

## Abstract (fr)

Les trypanosomes d’animaux sont potentiellement pathogènes pour l’homme. Plusieurs infections humaines par *Trypanosoma lewisi*, parasite du rat, ont été rapportées. Leur nombre est probablement sous-estimé. Certaines de ces infections auto-guérissent, d’autres nécessitent un traitement avec des médicaments utilisés dans la trypanosomose humaine africaine. Une évaluation *in vitro* de ces médicaments et du fexinidazole, un nouveau candidat-médicament actif par voie orale, a été réalisé sur *T. lewisi* en comparaison avec *T. brucei gambiense*. Tous ont présenté des effets comparables contre les deux parasites sauf la suramine qui n’était pas efficace. *In vivo*, ces médicaments ont été administrés à des rats immunodéprimés par le cyclophosphamide. La meilleure efficacité a été obtenue avec le fexinidazole et la pentamidine (15 mg/kg) : les rats ont été guéris en respectivement 7 et 10 jours. Ceux recevant la combinaison thérapeutique nifurtimox-éflornithine (NECT) ou pentamidine (4 mg/kg) ont été guéris au bout de 28 jours, tandis que le mélarsoprol était faiblement actif. L’identification de médicaments efficaces peu toxiques aidera à la prise en charge de nouveaux cas de trypanosomose atypique.

## Introduction

*Trypanosoma* (*Herpetosoma*) *lewisi* is an extracellular protozoan blood parasite of rats and is distributed worldwide [8]. It is naturally transmitted to rats by fleas. *T. lewisi* has a limited antigenic variation [6, 15]. In rats, *T. lewisi* infection is self-limited leading to elimination of circulating parasites and protection against re-infection. The typical pathogenic human trypanosomes are *T. brucei gambiense*, *T. b. rhodesiense* and *T. cruzi* [8]. Humans possess an innate protection against most trypanosomes species from animals [21]. However, trypanosomes from animals can in some cases be pathogens for humans. For instance, in 1933, a case of febrile Malaysian child presenting numerous *T. lewisi* parasites was reported [13]. In India, *T. lewisi* were detected in two adults [18] leading to self-cured while a baby was treated using pentamidine in 2010 [23]. These atypical human infections by animal trypanosomes were recently reviewed [20]. However, no evaluation of trypanocidal drugs, including the combination nifurtimox/eflornithine NECT [17], has been performed against *T. lewisi*. In this study, the efficacy of these drugs against *T. lewisi* was investigated *in vitro* and *in vivo* in cyclophosphamide (CPA)-treated and *T. lewisi*-infected rats [5]. The efficacy of Fexinidazole, a new oral nitroimidazole drug candidate for treatment of both stages in human African trypanosomosis (HAT), was also evaluated [19].

## Materials and methods

### Animals and ethics

Female Swiss mice, 18–20 g, and Wistar rats, 90–100 g (Charles River, L’Arbresle, France) were kept in our animal housing facility for more than a week before the experiment was started. Experiments on animals complied with guidelines of the European Convention for the Protection of Vertebrate Animals used for Experimental and other Scientific Purposes (CETS No. 123). Experiments were approved by the Department for the protection of animals and plants of the Préfecture de la Gironde (January 2012) including experiments on drug activity and toxicity (Identification number A33-063-324).

### Parasites

*T. b. gambiense* (Feo/ITMAP/1893) was used for *in vitro* experiment. Swiss mice were infected by intraperitoneal injection (IP) with 10^4^ parasites diluted in physiological saline.

*T. lewisi* (Wery L307 24/9/68), kindly provided by Étienne Pays and Pierrick Uzureau (Université Libre de Bruxelles, Gossselies, Belgium), was used for *in vitro* and *in vivo* experiments. Rats were infected by IP injection of 5 × 10^4^ parasites diluted in physiological saline.

Parasites were purified from rodent blood using DEAE-cellulose.

### Drugs

Current drugs used in HAT (pentamidine, suramin, melarsoprol, eflornithine, nifurtimox) were kindly supplied by World Health Organization. Fexinidazole was kindly supplied by Sanofi.

### *In vitro* assessment of drugs

Each well of a 96-well plate (Falcon Plastics, Oxnard, CA, USA) was filled with 100 μL of culture medium McCoy 5A modified medium supplemented with 100 U/mL penicillin, 100 μg/mL streptomycin, 25 mM HEPES, 0.1 mM 2-mercaptoethanol, 2 mM sodium pyruvate, 0.2 mM *L-*cysteine and 10% foetal calf serum [1, 24]. Nifurtimox and fexinidazole were first dissolved in 100% dimethyl sulphoxide (DMSO). Adequate dilutions in culture medium of each compound were added into each well in triplicate while control wells contained medium alone or with DMSO (0.4%). Then 100 μL of a suspension containing 10^5^ blood-purified parasites was added in each well. Cultures were maintained at 37 °C in 5% CO_2_ incubator for 24 h. Parasite count was performed using a haemocytometer. The activity was expressed in concentration inhibiting parasite growth by 50% (IC_50_) [16]. All experiments were performed twice, with each drug concentration in triplicate. The mean of IC50 and standard deviation (*SD*) obtained for the six cultures of each drug were calculated.

### *In vivo* assessment

Female rats were immunosuppressed by IP injection of CPA (100 mg/kg) 72 h before infection by IP injection of 5 × 10^4^ purified *T. lewisi* diluted in physiological saline.

On day 7 after infection, parasitemias were evaluated in blood collected by tail cutting [9]. Parasite counting was carried out using an haemocytometer for each rat, and six groups of seven rats were then randomly distributed:

- One group received pentamidine (4 mg/kg) by IP injection for 28 days;
- One group received pentamidine (15 mg/kg) by IP injection for 12 days;
- One group received three cures of melarsoprol (3.6 mg/kg/day) by IP injection, each cure is composed of 3 days spaced by a 7 day resting period;
- One group received fexinidazole (100 mg/kg) orally for 10 days;
- One group received eflornithine (200 mg/kg, twice a day) by IP injection and nifurtimox (10 mg/kg, twice a day) orally for 28 days;
- One group (control group) received physiological saline (0.5 mL/day) by IP injection for 28 days.

The number of parasites observed for each group was estimated as the mean of daily parasitaemia of the seven rodents.

### Statistical analysis

Parasitaemias are represented as the mean of the group ±*SD*. The comparisons between groups were made using the non-parametric Wilcoxon/Kruskal-Wallis test implemented in the JMP7 software.

## Results

### *In vitro* activity of drugs against trypanosomes

The reference drugs, pentamidine, suramin, melarsoprol, eflornithine and fexinidazole, were assessed for *in vitro* efficacy against trypanosomes. Their activity against *T. lewisi* and *T. b. gambiense* was compared. Pentamidine, eflornithine, nifurtimox and fexinidazole have comparable activities against the two parasites while melarsoprol is less active against *T. lewisi* than *T. b. gambiense* (Table 1). Suramin was not effective against the two parasites, and, due to its toxicity [22], suramin was not investigated further. The highest concentration of DMSO (0.4% in culture medium) had no trypanocidal activity.

**Table 1.** *In vitro* activities of trypanocidal drugs (IC_50_ (μM)).

	*T. lewisi*	*T. b. gambiense*
Melarsoprol	0.09 ± 0.01	0.03 ± 0.01
Pentamidine	0.07 ± 0.02	0.06 ± 0.02
Eflornithine	20.5 ± 8.7	25.1 ± 12.4
Nifurtimox	4.25 ± 1.06	3.12 ± 1.27
Fexinidazole	3.75 ± 0.85	2.55 ± 0.42

Each result is the mean ± *SD* of IC_50_ for six cultures.

### *In vivo* assessment of drugs

In rats infected by *T. lewisi* but not immunosuppressed by CPA, the blood parasite number was too low to allow a comparison between drugs (data not shown).

For rats which received CPA (100 mg/kg) 3 days before infection, drugs were given orally or by IP injection starting at day 7 after infection when parasite blood count was about 1.5 × 10^7^/mL.

**Figure 1.**
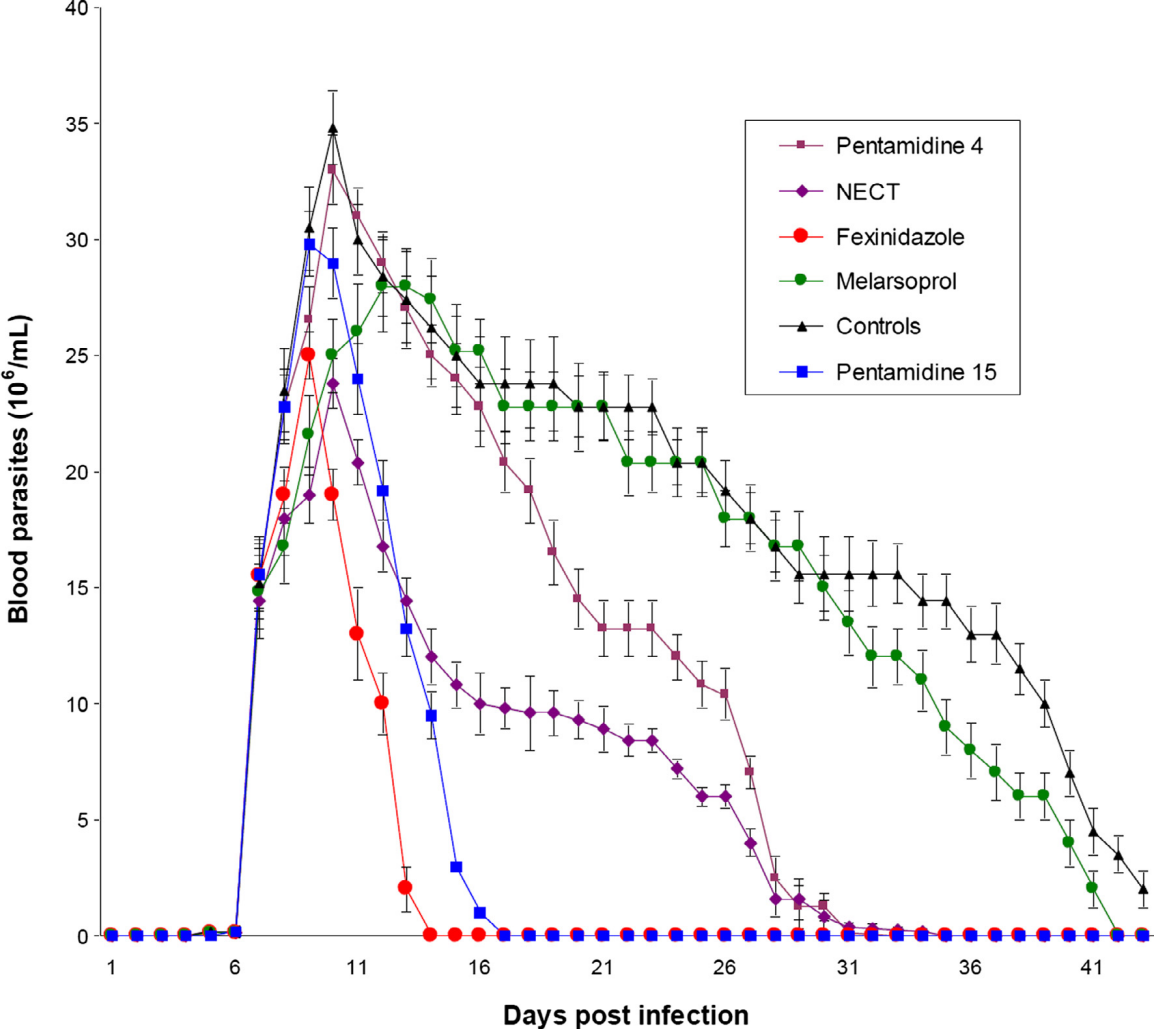
Evolution of parasitemias for *T. lewisi*-infected rats during 43 days after infection. Rats were infected by IP injection of 5 × 10^4^ purified *T. lewisi* and randomly divided in six groups at day 7 post infection. Each group received drugs as described. Parasitemias were monitored every day.

No animal died. In the control group (animals injected with physiological saline), a steady decrease of blood parasitaemia was observed after day 10. Nevertheless some animals remained infected over the all experimental period and still displayed parasitaemia levels (>10 × 10^6^ parasites/mL) 43 days after infection. As compared to controls, the control of parasitaemia was quicker for the animals of the fexinidazole and pentamidine (15 mg/kg) groups with all animals being cured respectively 7 and 10 days after treatment. The decay rate of blood parasitaemia was slower in the NECT and pentamidine (4 mg/kg) groups and all animals were cured after 33 and 35 days post infection respectively. Mean parasitaemia (parasites/mL) was however significantly lower (*p* = 0.006) in the NECT group (9 × 10^6^; calculated from day 7 to day 32 post infection) than in the pentamidine (4 mg/kg) group (15.8 × 10^6^; calculated from day 7 to day 34 post infection). The evolution of parasitaemia in the melarsoprol group was not significantly different from the controls until day 33 post infection and animals were finally cured at day 42 post infection although three animals of this group died during the experiment whereas no animal from others groups died. For each group, the sum of daily parasitaemia means (considered as whole parasite load and corresponding to area under the curves in Figure 1) ±*SD* was assessed from day 6 to day 43 post infection. The values were 724.36 ± 53 for control group, 103.76 ± 9.2 for fexinidazole group, 167.264 ± 11.1 for pentamidine (15 mg/kg) group, 252.318 ± 22.1 for NECT group, and 412.578 ± 28 for pentamidine (4 mg/kg) group. Compared to control group, parasite load was lower in all treated groups (*p* < 0.001). Compared to pentamidine (15 mg/kg) group, parasite load was lower in fexinidazole group (*p* < 0.05).

All treated rats remained negative in parasitology until 90 days post infection.

## Discussion

In this study, the efficacy of drugs for HAT treatment and fexinidazole against *T. lewisi* was demonstrated both *in vitro* and *in vivo*.

Melarsoprol and pentamidine have already been used successfully in two *T. lewisi* human infections [11, 23]. *In vitro* studies reveal a comparable susceptibility profile of *T. lewisi* and *T. b. gambiense*. Fexinidazole was both efficient against *T. lewisi* and *T. b. gambiense*. A single strain of *T. lewisi* was tested and other ones from various sources will be analysed very soon. However, fexinidazole possesses an *in vitro* trypanocidal efficiency on all tested *bruce*i subspecies, in the range of 0.7–3.3 μM and is also efficient on other parasites [2–30].

Rats were immunosuppressed using CPA at non-lethal doses to increase parasitemias [5]. In spite of heavy parasitaemia developed in CPA-treated rats, the brain remained free of parasites [5]. CPA mediates immunosuppression, which might mimic a potential but not established immunodeficiency in atypical human infections by animal trypanosomes. Some trypanocidal drugs require an intact immune system for clearance of trypanosomes [4] whereas nitroimidazoles are active compounds against various infectious agents, largely used and efficient in immunocompromised patients [10]. Fexinidazole and NECT were active at the doses recommended for HAT treatment but cure was obtained earlier with fexinidazole. However, fexinidazole may be active at lower doses and a further study will determine its minimum effective dose. Pentamidine was not active at the dose of 4 mg/kg, which is recommended in humans for African trypanosomiasis and *Pneumocystis jirovecii* pneumonia treatment. As superior doses exhibit activity in *Pneumocystis jirovecii*-infected rats [29], a 15 mg/kg dose was also assessed in *T. lewisi*-infected rats and was efficient.

Whereas human infections with *T. lewisi* can self-cure, it is not always the case [20]. Thus evaluating the efficacy of available drugs is of interest, in particular when *T. lewisi* are present in cerebrospinal fluid, as reported previously [11]. Fexinidazole and NECT are both efficient for patients in the neurological stage of HAT [19]. Compared to melarsoprol and eflornithine, NECT has a reduced toxicity and is much easier to administer [17]. Animal toxicology studies reveal that fexinidazole has an excellent safety profile [19]. A reduced toxicity of the drug, a better control of parasite number and persistence, a reduced production of TNF-*α* and reactive oxygen species by activated macrophages [25] might be beneficial in treatment by fexinidazole.

The number of atypical human infections attributable to primarily animal trypanosomes is possibly underestimated, mainly due to lack of tools and strategies to better detect infection [20]. Interaction with others factors might be involved in atypical human infections by animal trypanosomes. For instance, diet deficiency enhanced *T. lewisi* infections in rats, as also reported for the related murine parasite, *T. musculi* [12]. Moreover, immunomodulation of immune response might also be involved. The binding of immunoglobulins by parasites via receptors for their Fc region might cover parasites and decrease the efficiency of host immune response [3, 30]. The risk and potential impact related to atypical human infections by animal trypanosomes cannot be evaluated thoroughly at the present time and further studies are required. However the identification of drugs efficient against *T. lewis*i with reduced toxicity will help in the management of new cases. Fexinidazole and pentamidine (15 mg/kg) represent valuable drugs to treat *T. lewisi* infections.
